# Single- and Bicomponent Analyses of T2⁎ Relaxation in Knee Tendon and Ligament by Using 3D Ultrashort Echo Time Cones (UTE Cones) Magnetic Resonance Imaging

**DOI:** 10.1155/2019/8597423

**Published:** 2019-02-18

**Authors:** Jin Liu, Amin Nazaran, Yajun Ma, Haimei Chen, Yanchun Zhu, Jiang Du, Shaolin Li, Quan Zhou, Yinghua Zhao

**Affiliations:** ^1^Department of Radiology, Third Affiliated Hospital of Southern Medical University (Academy of Orthopedics, Guangdong Province), 183 Zhongshan Da Dao Xi, Guangzhou 510630, China; ^2^Department of Radiology, University of California, San Diego, San Diego, CA, USA

## Abstract

The collagen density is not detected in the patellar tendon (PT), posterior cruciate ligament (PCL), and anterior cruciate ligament (ACL) in clinic. We assess the technical feasibility of three-dimension multiecho fat saturated ultrashort echo time cones (3D FS-UTE-Cones) acquisitions for single- and bicomponent T2⁎ analysis of bound and free water pools in PT, PCL, and ACL in clinic. The knees of five healthy volunteers and six knee joint samples from cadavers were scanned via 3D multiecho FS-UTE-Cones acquisitions on a clinical scanner. Single-component fitting of T2⁎_M_ and bicomponent fitting of short T2⁎ (T2⁎_S_), long T2⁎ (T2⁎_L_), short T2⁎ fraction (Frac__S_), and long T2⁎ fraction (Frac__L_) were performed within tendons and ligaments. Our results showed that biexponential fitting was superior to single-exponential fitting in PT, PCL, and ACL. For knee joint samples, there was no statistical difference among all data in PT, PCL, and ACL. For volunteers, all parameters of bicomponent fitting were statistically different across PT, PCL, and ACL, except for T2⁎_S_, T2⁎_L_, and T2⁎_M_ resulting in flawed measurements due to the magic angle effect. 3D multiecho FS-UTE-Cones acquisition allows high resolution T2⁎ mapping in PT, PCL, and ACL of keen joint samples and PT and PCL of volunteers. The T2⁎ values and their fractions can be characterized by bicomponent T2⁎ analysis that is superior to single-component T2⁎ analysis, except for ACL of volunteers.

## 1. Introduction

Many of the degenerate tendons and ligaments from cadavers and biopsies from patients had a decreased collagen concentration and this change may predispose the tendons and ligaments to rupture, as a reduction in the collagen density has been correlated with the tensile strength of tendons and ligaments. If we can detect the reduction in the collagen density in the degenerate patellar tendon (PT), posterior cruciate ligament (PCL), and anterior cruciate ligament (ACL) in clinic, some medical methods might be used to prevent PT, PCL, and ACL from being ruptured. However, tendons and ligaments typically have very short transverse relaxation times (T2s or T2*∗*s) and therefore remain “invisible” with conventional clinical MRI sequences. As a result, early stages of tendon and ligament degeneration may not be detected by traditional MRI.

Ultrashort echo time (UTE) techniques, which use nominal TEs about 10-200 times shorter than those of conventional clinical MR sequences, can directly detect signal from short T2 tissues and might be used for diagnosis of these diseases at early stages [1, 2]. However, fat in the knee tissue can cause high levels of signal as well as artifacts like chemical shift artifact in cones trajectory-based UTE imaging [3]. Fat saturation (FS) techniques can be used to increase tissue contrast and to provide more accurate T2*∗* measurements [4]. Previous studies showed that at the early stages of ligaments and tendons degeneration, fat-suppressed UTE T2*∗* mapping could potentially reflect the biological composition and structural integrity in ligaments and tendons, which are important factors for detection of degeneration in the early stages [5].

Most knee joint tissues, including PT, PCL, and ACL, have two components, namely, bound water (BW) and free water (FW). Free water has a longer T2*∗* and is located between the network of interwoven collagen bundles, and bound water has a shorter T2*∗* and is associated with collagen and/or proteoglycan [2, 6–8]. Single-exponential calculation of T2*∗* values (T2*∗*_M_) alone is not able to discern short and long T2*∗* components. By performing bicomponent UTE T2*∗* analysis, “short” (T2*∗*_S_) and “long” (T2*∗*_L_) T2*∗* values and fractions can be determined. The short T2*∗* value, T2*∗*_S_, represents bound water while the long T2*∗* value, T2*∗*_L_, represents free water [9, 10].

However, bicomponent analysis typically requires a long scan time to allow acquisition of all images at different TEs [9, 11]. High spatial resolution is also needed in order to image the fine structures in the knee joint. As a result, the in vivo application of the bound and free water mapping techniques is still limited [12–14]. So we had a hypothesis that the bicomponent analysis allowed the in vivo application of the bound and free water mapping techniques for PT, PCL, and ACL using 3D multiecho fat saturated ultrashort echo time Cones (3D FS-UTE-Cones) imaging protocol in clinic. This investigation would provide for clinical doctors with a method to detect the early degeneration of PT, PCL, and ACL. In this study, we aimed to assess 3D multiecho fat saturated ultrashort echo time Cones (3D FS-UTE-Cones) imaging protocol for single- and bicomponent T2*∗* mapping of free and bound water components for PT, PCL, and ACL in a clinical 3T scanner.

## 2. Materials and Methods

### 2.1. Data Acquisition Methods

Five heathy volunteers (4 males, aging from 25 to 30; one female, 46 years old) were enrolled to investigate the clinical feasibility of 3D multiecho FS-UTE-Cones imaging techniques using a clinical whole-body 3T scanner. Written informed consent and approval from the institutional review board (IRB) of our hospital were obtained before the in vivo scans. The inclusion criteria for the volunteers were as follows: no history of knee joints pain, no nontraumatic joint pain history, and no metal implants or pacemakers.

Six sets of PCL, ACL, and PT samples from cadaveric knees of six donors (2 males, 4 females, age range = 24–65 years, and mean ± standard deviation of 47.5 ± 14.5 years) were obtained from University California, San Diego morgue. A transverse cut at the proximal one-third of the samples and a longitudinal cut through the center of the ligament stored in -20°C refrigerator. A transverse slab of ~10 mm thickness and a longitudinal slab of ~5mm thickness were cut and stored in a phosphate buffered saline (PBS) soaked gauze at 4°C prior to MR imaging. After the ex vivo scans, the samples from the center of the PCL, ACL, and PT substance were immediately fixed in Z-Fix (Anatech, Battle Creek, MI) for histology. Samples were embedded in paraffin, and five micrometer thick sections were cut and stained with hematoxylin and eosin (H&E).

**Figure 1:**
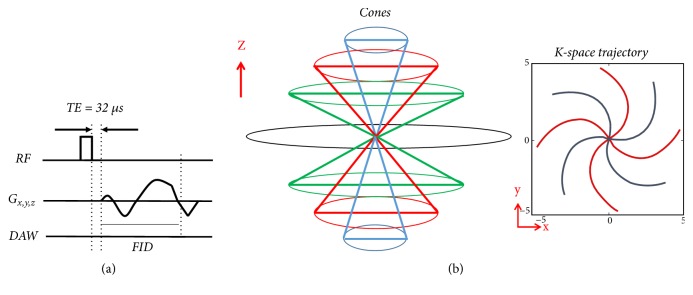
The 3D UTE Cones sequence (a). After excitation with a short rectangular pulse, a 3D Cones trajectory (b) is used to allow time-efficient sampling with a minimal TE of 32 *μ*s.

An 8-channel transmit-receive knee coil and a 3-inch coil was used for all volunteer and cadaveric samples acquisitions, respectively. The 3D FS-UTE-Cones sequence employs a short rectangular pulse excitation (pulse duration = 32 *μ*s) followed by 3D spiral trajectories with conical view ordering (Figure 1). The sequence allows anisotropic resolution (e.g., high in-plane resolution and thicker slice) for much-improved signal-to-noise ratio (SNR) and reduced scan time.

To save scan time, a multiecho FS-UTE-Cones acquisition scheme was designed for mapping of T2*∗* relaxation times. For knee joint samples, the acquisition parameters were TR = 48 ms, four groups of four echoes (TE (0.2/3.3/15 ms, 0.5/5.5/20 ms, 0.8/8/25 ms, 2.1/11/30 ms), flip angle (FA) = 16°, bandwidth (BW) = 128 kHz, field of view (FOV) = 8×8cm, acquisition matrix of 256×256, 26 slices with a slice thickness of 2 mm, and a total scan time of 12 minutes. In clinic, except for the same acquisition matrix with knee joint samples, the acquisition was TR = 86.2 ms, four groups of four echoes were TEs = 0.032/4.4/20/40 ms, 0.4/6.6/25/50 ms, 0.8/1/30/60 ms, and 2.2/16/35/70 ms, flip angle (FA) = 14°, bandwidth (BW) = 125 kHz, field of view (FOV) = 20×20 cm, 30 slices with a slice thickness of 4 mm, and total scan time of 18 minutes. Image of axial level is obtained.

### 2.2. Definition of Region of Interests (ROIs)

**Figure 2:**
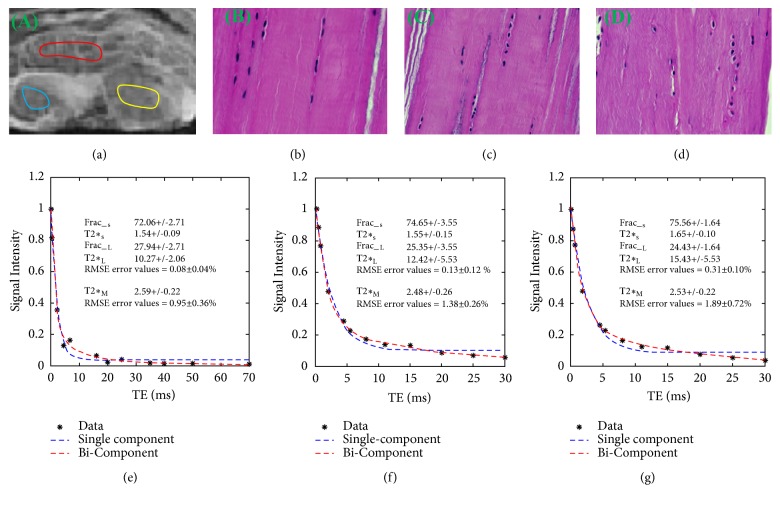
Selected 3D UTE Cones images and region-of-interest (ROI) shown in a patella tendon (PT) sample with red lines, a posterior cruciate ligament (PCL) sample with yellow lines, and anterior cruciate ligament (ACL) sample with blue lines (a), followed by histology in the ROI of the PT (b), PCL (c), and ACL (d), where collagen is arranged in tightly cohesive well-demarcated bundles (stain: hematoxylin and eosin; original magnification, *∗*200), as well as single- and bicomponent fitting (e, f, g) of interleaved multiecho UTE image acquired at TE (0.2/3.3/15 ms, 0.5/5.5/20 ms, 0.8/8/25 ms, and 2.1/11/30 ms of a 45-year-old female cadaver). All bicomponent fitting shows superior over single-component fitting. Dashed lines represent the estimated T2*∗* curve and solid black circles represent the data points.

**Figure 3:**
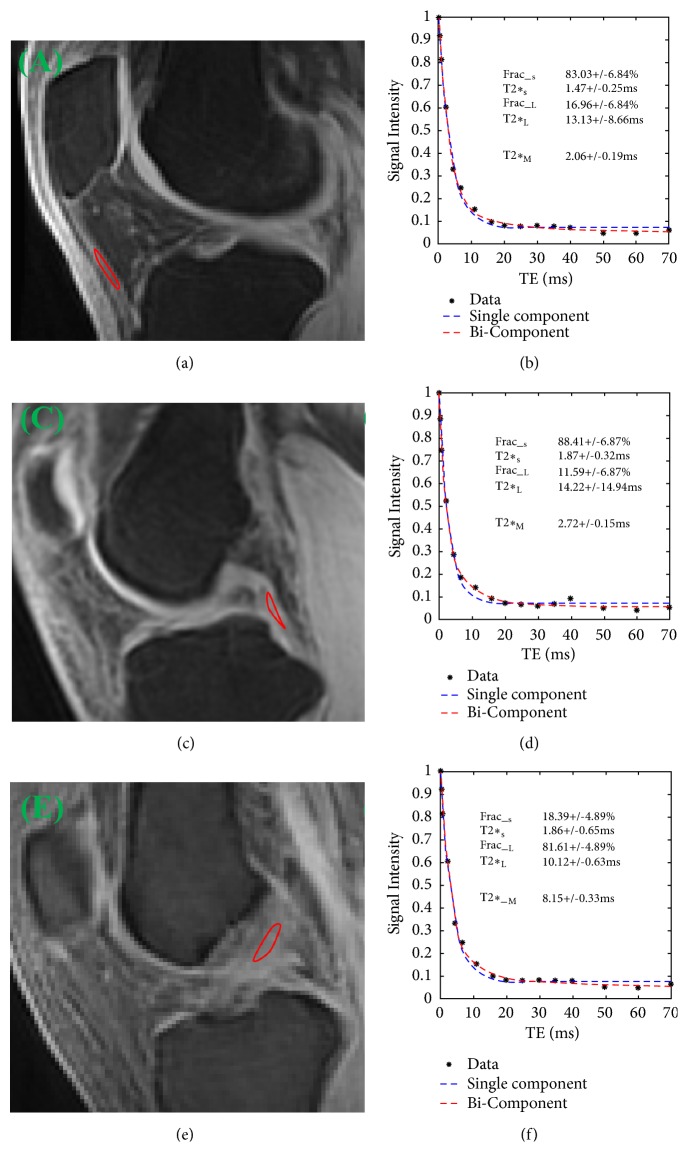
Selected 3D UTE Cones images and region-of-interest (ROI) shown with red lines in patella tendon (PT) (a), posterior cruciate ligament (PCL) (c), and anterior cruciate ligament (ACL) (e), as well as single- and bicomponent fitting (b, d, f) of interleaved multiecho UTE image acquired at TE (0.032/4.4/20/40 ms, 0.4/6.6/25/50 ms, 0.8/1/30/60 ms, and 2.2/16/35/70 ms of a 29 years old male volunteer). All bicomponent fitting shows superior over single-component fitting. Dashed lines represent the estimated T2*∗* curve and solid black circles represent the data points.

Both single- and bicomponent T2*∗* analyses were performed in MATLAB (The Math Works Inc., Natick, MA, USA) using code developed in-house as previously described [15]. The ROIs were drawn on PT, PCL, and ACL in volunteer knee joints and samples, respectively. The maximum areas of PT, PCL, and ACL were obtained. To minimize partial volume effects, the following criteria were taken into account during the selection of ROIs. First, the middle slice was chosen for the PT, PCL, and ACL analyses. Second, the ROIs were at least 1.0 mm away from articular cartilage. Third, the ROIs were placed near the inner edge of PT, PCL, and ACL. For samples, ROIs were drawn in the middle of PT, PCL, and ACL. As shown in Figures 2 and 3, from which the average signal was used for fitting. Mean UTE-T2*∗* values for ROIs were recorded for analysis and evaluated by a musculoskeletal radiologist of twenty- year experiences.

### 2.3. Analysis of Single- and Bicomponent Fitting

Both single- and biexponential fitting procedures were performed on the selected ROIs, for all MR data sets. For single-exponential fitting, a three-parameter function (see (1)) was used to fit the signal intensity where S_N_(t) is the signal intensity, and A is the amplitude of the total component T2*∗*_M_.

(1) $$\begin{array}{c} SN\left(\;t\;\right)=A\times \exp\left(\;-\frac{t}{T2{*}_{M}}\;\right)+noise \end{array}$$

The same data set was fitted biexponentially based on the following equation:

(2) SNt=As×exp⁡−tT2∗S+AL×exp⁡−tT2∗L+noise

A_S_ is the amplitude of the short component, A_L_ is the amplitude of the long component, T2*∗*_S_ is the short component T2*∗*, and T2*∗*_L_ is the long component T2*∗*. Apparent short component fraction (Frac_s) was defined as A_S_/(A_S_ + A_L_), and long component fraction (Frac__L_) was defined as A_L_/(A_S_ + A_L_).

For the T2*∗* calculation, only the pixels that satisfied the following condition (3) were considered for the biexponential fitting:

(3) $$\begin{array}{c} 4\times T_{2*S}<T_{2*L} \end{array}$$

Background noise was determined using maximum likelihood estimation (MLE) distribution fitting of a partial histogram. Nonnegative least square curve fitting was used for both single- and bicomponent models. Fitting curves along with 95% confidence intervals (CI) and residual signal curves were created [16]. The root means squared error (RMSE) was calculated to quantify the goodness of fits [17].

As a result, the bicomponent fitting model can estimate T2*∗*s and fractions of the two components with a root-mean-square error (RMSE) value of less than 3%, providing a clinically achievable SNR of 60 or higher.

### 2.4. Statistics

All statistical analyses were analyzed in SPSS Statistics version 13.0 for Windows. Calculated values, including T2*∗*_M_, T2*∗*_S_, T2*∗*_L_, Frac_s, Frac__L_, and RMSE, were described as the means and standard deviation (SD) for normal PT, PCL, and ACL. An independent sample T-test with equal variances was performed to obtain the difference in RMSE between single- and bicomponent analysis of PT, PCL, and ACL, respectively. Pairwise differences across T2*∗*_M_, T2*∗*_S_, T2*∗*_L_, and their fractions were also analyzed for PT, PCL, and ACL.* P* less than 0.05 were considered statistically significant.

## 3. Results

### 3.1. RMSE Analysis for Single- and Biexponential Fitting Model

Table 1 represents the RMSE error for the single- and bicomponent T2*∗* analysis. RMSE error values (means ± SD) of bi- versus single-exponential fitting model were shown as follows: for knee joint samples, 0.08 ± 0.04% versus 0.91 ± 0.41% for PT; 0.12 ± 0.02% versus 1.38 ± 0.25% for PCL; 0.33 ± 0.09% versus 1.91 ± 0.71% for ACL; for volunteers, 1.63 ± 0.15% versus 2.56 ± 0.12% for PT; 1.34 ± 0.13% versus 2.57 ± 0.25% for PCL; 3.23 ± 0.33% versus 3.62 ± 0.33% for ACL. Biexponential fits were significantly different from single-exponential fits for PT and PCL (P = 0.01 in both cases), while there was no statistical significance between the single- and biexponential fits for ACL (P = 0.29) in volunteer. The single- and biexponential fits in ACL had the largest errors among RMSEs in PT, PCL, and ACL in volunteer.

Figure 2 shows that RMSE error values of bicomponent fitting model are similar to ones of single-component fitting model (0.08 ± 0.04% versus 0.95 ± 0.36% for PT; 0.13 ± 0.12% versus 1.38 ± 0.26% for PCL; 0.31 ± 0.10% versus 1.89 ± 0.72% for ACL) in knee joint samples.

Simulation results are shown in Figure 3, where there is excellent bicomponent with much smaller fitting error than single-component in PT (1.51 ± 0.13% versus 2.06 ± 0.19%) and PCL (1.26 ± 0.12% versus 2.43 ± 0.27%). However, the fitting errors of single- and bicomponent fitting are very large in ACL (3.25 ± 0.31% versus 3.42 ± 0.38%) in healthy knees and as was true of the pooled data.

### 3.2. Analysis of UTE-T2*∗* Values of PT, PCL, and ACL

Table 2 summarizes both single- and bicomponent analyses of PT, PCL, and ACL of all six samples and five volunteers. For samples, T2*∗*_S_, T2*∗*_L_, Frac__S_, Frac__L_, and T2*∗*_M_ obtained in PT are similar to all data measured in PCL and ACL, which have been proved to be normal tissues using histology. Figure 2 example of knee joint samples shows that the fibers were arranged close and parallel to each other with slight waviness in normal PT, PCL, and ACL.

However, for volunteer, T2*∗*_S_, T2*∗*_L_, Frac__L_, and T2*∗*_M_ are smaller in PT and PCL than those in ACL. On the contrary, Frac__S_ was smaller in ACL than in PT and PCL (90.54 ± 2.69% in PT, 87.02 ± 3.85% in PCL, and 21.91 ± 9.05% in ACL) (Figure 3).

**Figure 4:**
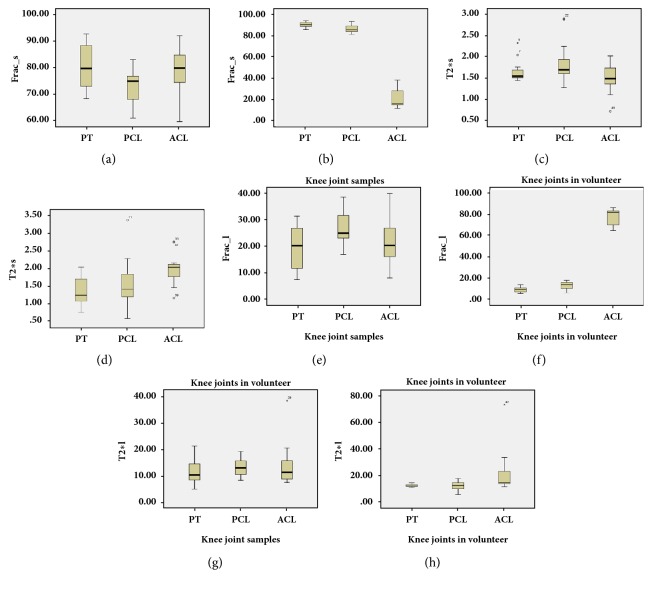
Box plots of Frac__s_ (a, b), T2*∗*s (c, d), Frac__s_ (e, f), and T2*∗*L (g, h) in patellar (PL), posterior cruciate ligament (PCL), and anterior cruciate ligament (ACL) of samples ((a), (c), (e), and (g)) and volunteer ((b), (d), (f), and (h)). Top and bottom of boxes represent 25–75 percentiles of the data values. The line in the box represents median value.

Table 3 shows results from independent sample T-tests. Under the condition of P values < 0.05 indicating significant difference, for samples, all measurements from bicomponent model are not different than all data from single-component model (all* P* > 0.05); for volunteers, there are significant differences in Frac__S_ and Frac__L_, for PT versus PCL, for PCL versus ACL, and for PT versus ACL, with the *P* value of 0.01 for all cases. Moreover, T2*∗*_S_, T2*∗*_L_, and T2*∗*_M_ show statistical differences between PCL and ACL and between PT and ACL.* P *values for all the aforementioned cases were 0.01. On the contrary, no significant differences were found for T2*∗*_S_, T2*∗*_L_, or T2*∗*_M_ between PT and PCL (*P *= 0.63;* P* = 0.88;* P *= 0.05, respectively) or between PCL and ACL (*P* = 0.47;* P* = 0.41;* P* = 0.06, respectively).  Figure 4 shows box plots of Frac_s (A), T2*∗*s (B), Frac__L_ (C), and T2*∗*_L_ (D) in PT, PCL, and ACL of samples and volunteers. Except for measurements in ACL of volunteers, variations among all data were smaller. If we applied 0.01 < P values < 0.001, there was no statistic significance among all measurements.

## 4. Discussion

### 4.1. The Technical Feasibility of Multiecho 3D UTE Cones Acquisitions for Quantifying Knee Joints in Clinical Trials

Our results suggest that the 3D Cones FS-UTE sequences, together with an interleaved multiecho acquisition strategy, allow mapping of bound and free water T2*∗*s and relative fractions in PL, PCL, and ACL. Previously, 2D UTE sequences have been employed for bicomponent analysis of bound and free water components in various knee joint tissues [14]. 3D UTE Cones sequences have many advantages over 2D UTE sequences. 3D UTE cones sequences are much less prone to eddy current artifact compared with 2D UTE sequences with half-pulse excitation, where mapping of bound and free water components may suffer from errors due to out-of-slice signal contaminations [18]. In addition, 3D Cones UTE sequences have more desirable properties for UTE MRI than 3D projection reconstruction (3DPR), such as higher SNR efficiency, less aliasing artifacts, and reduced scan time [19, 20]. The high SNR efficiency of 3D UTE Cones imaging (Figure 2) allows robust mapping of the shorter T2*∗* and longer T2*∗* components [21]. Finally, the interleaved multiecho acquisitions in these sequences allow relatively short scan times of 18 minutes for volumetric coverage and high resolutions mapping of bound and free water T2*∗*s and relative fractions.

As shown in Table 1, we achieved biexponential T2*∗* fitting performance with very small RMSE values in all measurements, except for that in ACL of volunteers. This is consistent with the previous results published by Raya et al., showing that biexponential analysis can reduce errors to less than 4% with an SNR of 50 [22]. Single- and biexponential T2*∗* fitting in ACL is inferior to that in PT and PCL. These results might be explained by the fact that ACL is oriented closer to the magic angle (~54°) relative to the B0 field and thus exhibits greatly reduced the signal from short T2 components.

### 4.2. Single-Component Fitting of T2*∗* Values in PT and Ligaments

The T2*∗*_M_ of 2.05 ± 0.27 ms in PT was consistent with the range of values from healthy volunteers shown by Kijowski et al. via a UTE-T2*∗* mapping sequence in a 3.0T scanner (T2*∗*_M_ of 2.0 ms, 95% CI 1.5–2.4 ms) [2]. However, there have been no studies of T2*∗*_M_ in ligament using 3D UTE before. Our preliminary results show that the T2*∗*_M_ in PT were similar to ones in PCL and ACL of knee joint samples, and that the T2*∗*_M_ was lower in PT (2.05 ± 0.27 ms) than in PCL (2.21 ± 0.96 ms), and much lower than in ACL (7.65 ± 1.29 ms) of volunteers. It is well known that the percent of water in PT is known to be 60-70%, which is similar to the 55–65% percent water in ligament [23]. Additionally, the dry weight of normal PT, PCL, and ACL consists of 65–80% collagen (mostly type I) and 1–3% proteoglycan [24]. However, the bundles of collagen fibers are more parallel in PT than in ligament [25]. T2*∗* values can be influenced not only by the water and collagen content but also the collagen fiber orientation, including longitudinal, transverse, and oblique directions [24, 26]. In particular, previous studies have shown that small changes in the orientation of tissue relative to the direction of the main magnetic field can greatly alter the observed measurements of T2*∗* values [27, 28]. So, these studies might explain the higher T2*∗*_M_ in ACL than in PT and PCL of volunteers.

### 4.3. Bicomponent Fitting of T2*∗* Values in PT and Ligaments

Our results for T2*∗* bicomponent analyses in PT are consistent with results reported in previous studies. An example is a study performed by Kijowski et al., with T2*∗*_S_ of 1.5 ms (CI: 1.3–1.8 ms) and Frac__S_ of 75.5% (CI: 74.7–78.9%) in PT [2]. Similarly, Chang et al. used normal tendon samples and found T2*∗*_S_ of 1.8 ms (range 1.4-2.4 ms), Frac__S_ of 79% (CI: 67-93%), and Frac__L_ of 21% (range 8-33%) via 2D UTE sequences on a 3T MR scanner [5]. Our results are also consistent with those of Juras et al., wherein 10 healthy volunteers showed a Frac__S_ range from 47% to 79% and a T2*∗*_L_ range from 7.9 ms to 31.8 ms in PT. However, T2*∗*_L_ values in our study were intermediate between the T2*∗*_L_ of 23.1 ms (95%CI 21.7–25.0ms) reported by Kijowski et al. and the T2*∗*_L_ of 9.2 ms (range 5.6-16.4 ms) identified by Chang et al. This could be explained by the fact that collagen fiber organization affects the T2*∗*_L_ more than the T2*∗*_S_ in the knee. Also, measurements are susceptible to nearby tissues (such as fat and water in knee joints), which result in lengthened T2*∗*_L_ in PT [29].

For the first time, we performed T2*∗* bicomponent analysis of PCL and ACL by using a 3D Cones UTE sequence. Our study showed that all parameters were statistically different among PT, PCL, and ACL, except for T2*∗*_S_ and T2*∗*_L_ in PT versus PCL, as well as in PCL versus ACL of volunteers. The differences in parameters between PT and PCL were less than those between PT and ACL of volunteers in our results (Figure 3). Frac__S_ was higher in PT and PCL than in ACL of volunteers. The reason might be that the geometric arrangement of ACL resulted in flawed measurements due to the partial volume effect. ACL is thinner than PT and PCL, with the posterolateral (PL) and anteromedial (AM) bundles mean diameter of 9 mm (range 7–17 mm). For knee joint samples, collagenous fibers are parallel to the B0 field; there was no magic angle effect on the measurement in PT, PCL, and ACL. In addition, the relationship between magnetization fractions and matrix components may be affected by proton exchange between compartments. For example, Lattanzio et al. used a four-spin component exchange model in their study of articular cartilage [30]. A magnetization exchange rate of 120 s^−1^ between PG and collagen was found, which is intermediate to the corresponding nominal relaxation rates in our analysis of 1/T_2,1_ = 438 s^−1^ and 1/T_2,2_ = 39.6 s^−1^. Therefore, the T2*∗*s and associated fractions measured in our experiment might be influenced by this exchange. Moreover, as discussed by Zheng et al., T2*∗* measurements could actually reflect a combination of molecular, structural, and procedural levels of complications [31]. Hence, the signal from the PCL and ACL of volunteers might be complicated by their orientations relative to B0, response to fat suppression methods, the magic angle effect, etc. [32, 33].

In summary, our results suggest that multiecho 3D UTE Cones acquisitions have some advantages over existing technologies. First, the multiecho 3D FS-UTE-Cones acquisition allows highresolution 2D T2*∗* mapping. Volumetric analysis of T2*∗* mapping would be for future study. Second, bicomponent T2*∗* analysis can characterize the short and long T2*∗* values and fractions for PT and ligaments. It seems likely that both water bound to collagen and water bound to proteoglycans contribute to the short T2*∗* signal component. In particular, the short T2*∗* signal component is mainly derived from the water bound to collagen. On the contrary, the long T2*∗* signal component derived from the free water in ligament and tendon. So, the T2*∗*S and their fractions might provide information about water bound to collagen matrix, which would be used as biomarkers for early degeneration associated with injury of collagen matrix in PT and ligaments. 3D UTE Cones MR acquisition might be particularly useful for measurement of PT and PCL tissues, the distinction between abnormal from normal tissue. Finally, FS-UTE 3D Cones imaging can provide relatively shorter the total scan time of 18 min than UTE imaging in previous studies.

Our study has several limitations. First, 3D UTE requires longer scan times. The increased likelihood of patient movement increases susceptibility to motion artifacts and could introduce errors in biexponential T2*∗* mapping. Movement of the subjects was minimized by careful knee fixation, and the images were coregistered in postprocessing. Second, the number of volunteers was small, consisting of five knees from five healthy volunteers. With more volunteers, we expect that the RMSE of ACL would be decreased, that the difference between ALC and PCL would reach significance, and that clinical diagnostic guidelines for making decisions about disorders of PT, PCL, and ACL would be found using 3D UTE Cones MR acquisition. Finally, although the total scan times of 18 min were shorter than the scan times using UTE techniques in previous studies, in clinic MRI, this UTE scan time is still too long for clinical use. Further reduction in the total scan time will be explored in future studies via few TE, parallel imaging, and/or compressed sensing techniques.

## 5. Conclusions

This study confirms that interleaved multiecho 3D UTE Cones acquisitions allow T2*∗* mapping in a clinical setting. For PT and PCL, the short and long T2*∗* components and their fractions can be characterized by bicomponent T2*∗* analysis, which is superior to single-component analysis, having reduced RMSE during fitting and greater information on both bound and free water components.

## Figures and Tables

**Table 1:** T-test of RMSE between bi- and single-component.

Parameters	Knee joint samples (RMSE)			Knee joints in Volunteer (RMSE)		
	Bi-component (%)	Single-component (%)	P values	Bi-component (%)	Single-component (%)	P values
PT	0.08±0.04	0.91±0.41	0.01	1.63±0.15	2.56±0.12	0.01
PCL	0.12±0.02	1.38±0.25	0.01	1.34±0.13	2.57±0.25	0.01
ACL	0.33±0.09	1.91±0.71	0.01	3.23±0.33	3.62±0.33	0.29

*P* values < 0.05 indicate significant difference; *RMSE* root means square error; *PT*: patella tendon; *ACL*: anterior cruciate ligament; *PCL*: posterior cruciate ligament.

**Table 2:** Bi- and single-component T2*∗* analysis result for knee joint samples and healthy volunteers at PT, PCL, and ACL.

parameters	Samples				Volunteer		
	PT		PCL	ACL	PT	PCL	ACL
Bi-component	Frac__S_	80.44±8.31	75.50±6.49	79.24±7.43	90.54±2.69	87.02±3.85	21.91±9.05
	T2*∗*_S_	1.53±0.31	1.86±0.45	1.63±0.31	1.40±0.50	1.56±0.71	2.01±0.45
	Frac__L_	19.56±8.31	24.50±6.49	21.30±7.44	9.45±2.69	13.04±3.91	78.62±8.17
	T2*∗*_L_	11.83±4.63	13.53±3.46	13.58±6.59	12.96±1.08	12.81±3.47	13.08±3.38
Single-component	T2*∗*_M_	2.17±0.49	2.64±0.46	2.18±0.49	2.05±0.27	2.21±0.96	7.65±1.29

SD: standard deviation; PCL: posterior cruciate ligament; ACL: anterior cruciate ligament; PT: patellar tendon.

**Table 3:** *P* values of independent-samples T-test for bi- and single-component T2*∗* analysis results of knee joints sample and five healthy volunteers, at PT, PCL, and ACL.

parameters	Sample				Volunteer		
	PT_PCL		PCL_ACL	PT_ACL	PT_PCL	PCL_ACL	PT_ACL
Bi-component	Frac__S_	0.24	0.73	0.45	0.01	0.01	0.01
	T2*∗*_S_	0.21	0.28	0.46	0.63	0.47	0.01
	Frac__L_	0.24	0.69	0.47	0.01	0.01	0.01
	T2*∗*_L_	0.28	0.18	0.61	0.88	0.41	0.04
Single-component	T2*∗*_M_	0.58	0.23	0.06	0.05	0.06	0.01

PCL: posterior cruciate ligament, ACL: anterior cruciate ligament, and PT: patella tendon. P values < 0.05 indicate significant difference; 0.01 < P values < 0.001 are highly statistically significant.

## Data Availability

The data used to support the findings of this study are available from the corresponding author upon request.
